# Plasma Epstein-Barr Virus-Deoxyribonucleic Acid Copy Number Predicts Disease Progression in Stage I–III Pulmonary Lymphoepithelioma-Like Carcinoma

**DOI:** 10.3389/fonc.2020.01487

**Published:** 2020-08-21

**Authors:** Qi-Wen Li, Bo Qiu, Wan-Ming Hu, Su-Ping Guo, Ying-Jia Wu, Yu-Jia Zhu, Nan Hu, Xin-Lei Ai, Nai-Bin Chen, Jin-Yu Guo, Yong-Hong Hu, Meng-Zhong Liu, Mu-Sheng Zeng, Hui Liu

**Affiliations:** ^1^State Key Laboratory of Oncology in South China, Department of Radiation Oncology, Collaborative Innovation Center for Cancer Medicine, Sun Yat-sen University Cancer Center, Guangzhou, China; ^2^State Key Laboratory of Oncology in South China, Department of Pathology, Collaborative Innovation Center for Cancer Medicine, Sun Yat-sen University Cancer Center, Guangzhou, China; ^3^State Key Laboratory of Oncology in South China, Guangdong Key Laboratory of Nasopharyngeal Carcinoma Diagnosis and Therapy, Department of Experimental Research, Collaborative Innovation Center for Cancer Medicine, Sun Yat-sen University Cancer Center, Guangzhou, China

**Keywords:** pulmonary lymphoepithelioma-like carcinoma, Epstein-Barr virus, biomarker, tumor progression, monitoring

## Abstract

**Purpose:** To investigate the predictive values of plasma Epstein-Barr Virus (EBV)- deoxyribonucleic acid (DNA) copy number on disease progression and survival in stage I-III pulmonary lymphoepithelioma-like carcinoma (LELC).

**Patients and Methods:** Patients with pathologically confirmed, initially diagnosed or locally recurrent stage I-III pulmonary LELC, who received locally radical treatment and had plasma EBV-DNA results, were retrospectively reviewed. Risk factors of progression-free survival (PFS) and overall survival (OS) were assessed, including the predictive value of pre- and post-treatment EBV-DNA levels. The EBV-DNA change during follow-up was analyzed to determine its association with tumor progression and survival.

**Results:** A total of 102 patients were included in analysis. Eighty-eight patients had initially-diagnosed and 14 had locally recurrent disease. There were 33 patients treated with radical surgery, 55 with definite radiotherapy and 14 with both. EBV-DNA was tested pre-treatment (*N* = 66), post-treatment (*N* = 93) and/or during follow-up (*N* = 58). Forty-one patients had complete EBV-DNA results of all three time points. The overall 2-year PFS and OS were 66.3 and 96.0%, respectively. Pre-treatment EBV-DNA copy number > 10,000 copies/mL was a risk factor of PFS (2-year PFS, > 10,000 vs. ≤ 10,000 copies/mL, 37.2 vs. 75.1%, *p* = 0.007). Positive post-treatment EBV-DNA also indicated a worse PFS in univariable (2-year PFS, > 0 vs. 0 copy/mL, 25.6 vs. 76.8%, *p* < 0.001) and multivariable analysis (HR = 3.44, 95% CI, 1.52–7.78; *p* = 0.003). In the follow-up set, an increasing EBV-DNA exceeding 1,000 copies/mL strongly predicted disease progression within 3 months, with a specificity of 97.5% (95% CI: 86.8–99.6%) and was associated with impaired OS (2-year OS, > 1,000 vs. ≤ 1,000 copies/mL, 72.9 vs. 100%, *p* < 0.001).

**Conclusions:** Regular testing of EBV-DNA is suggested for pulmonary LELC to predict disease progression. If EBV-DNA copy number was increasing and beyond 1,000 copies/mL during follow-up, intensive radiologic evaluations are recommended.

## Introduction

**Figure 1:**
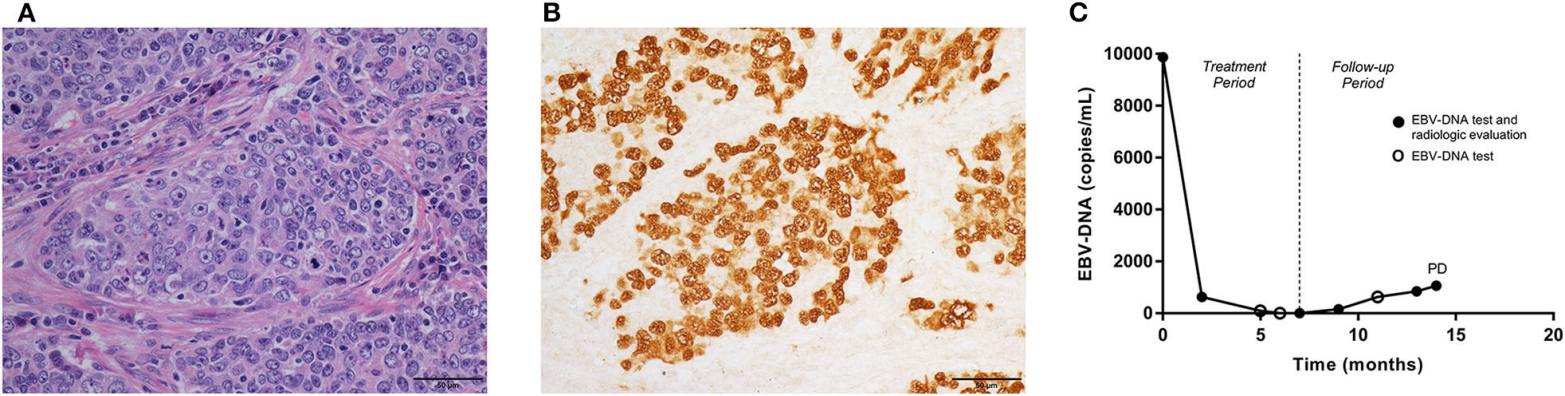
Pathologic features, the fluctuations of EBV-DNA copy number and disease development in one pulmonary LELC case. A 60-year-old, male patient was diagnosed with stage IIIA pulmonary LELC. He received radical surgery, adjuvant chemotherapy, and post-operative radiotherapy. Using the surgical specimen, **(A)** the Hematoxylin-eosin (HE) staining (×400) showed epithelial tumor cells nests surrounded and infiltrated by lymphocytes. **(B)** EBERs (×400) were detected by *in situ* hybridization in tumor cells. **(C)** The patient had pre-treatment EBV-DNA concentration of 9,870 copies/mL, and it decreased to zero after multimodality therapy. The EBV-DNA copy number had a slow increase during follow-up, but no trace of recurrence was found within the first 13 months since start of treatment. Once EBV-DNA copy number exceeded 1,000 copies/mL (1,060 copies/mL) at 14th months, a solitary metastasis in the liver was observed simultaneously by computed tomography. EBV, Epstein-Barr virus; DNA, deoxyribonucleic acid; LELC, lymphoepithelioma-like carcinoma; EBERS, EBV-encoded small non-polyadenylated RNAs; PD, progressive disease.

Pulmonary lymphoepithelioma-like carcinoma (LELC) is a rare subtype of non-small cell lung cancer (NSCLC), accounting for <1% of all histologic type of lung cancers. So far only ~600 cases have been reported in the English-language literature (1–7). Histologically, the disease is characterized by epithelial tumor cells nests surrounded or infiltrated by numerous lymphocytes (1) (Figure 1A). The disease is more prevalent in Asia, mostly affecting young, non-smoking patients (2, 3). The treatment principles for pulmonary LELC were not different from those of other NSCLCs, but with a more promising prognosis. Surgery, with or without adjuvant chemotherapy, resulted in a satisfactory survival rate (36-month recurrence-free survival, 73%) in resectable cases (6). In stage III–IV disease, a median overall survival time of more than 3 years is reported after multimodality therapy (7). There is growing evidence demonstrating an active role of Epstein-Barr virus (EBV) in the carcinogenesis and development of pulmonary LELC. Over 90% of pulmonary LELC cases presented positive EBV-encoded small non-polyadenylated RNAs (EBERs) in tumor cells, as a result of *in situ* hybridization (Figure 1B) and immunohistochemistry techniques. In some patients, the antibodies to the Epstein-Barr antigens, including latent membrane protein 1 and viral capsid antigen, were also detectable (8). In contrast, other lung carcinomas often showed signals of neither EBERs, nor latent membrane protein 1 or viral capsid antigen (9). Plasma EBV- deoxyribonucleic acid (DNA) is usually positive in patients with pulmonary LELC (4).

Based on the experience on nasopharyngeal cancer, the potential diagnostic, prognostic and predictive role of plasma EBV-DNA copy number in pulmonary LELC triggered much interest. Because of the rarity of pulmonary LELC, previous studies usually focused on small, heterogeneous cohorts with different tumor stage and inconsistent treatments (2–4). There lacks a universally accepted threshold of EBV-DNA copy number suggesting immediate disease progression for the patients after radical treatment. In this study, we retrospectively observed patients with stage I-III pulmonary LELC, who received radical treatment for initial or locally recurrent disease. Then we analyzed the prognostic effects of plasma EBV-DNA copy number, both pre- and post-treatment, on survival outcomes, as well as the association between sequential EBV-DNA copy numbers and tumor relapse during follow-up.

## Materials and Methods

### Study Population

The study was reviewed and approved by the ethics committee of Sun Yat-sen University Cancer Center. Because it was a retrospective study presenting anonymous data, we required and were granted a waiver of written informed-consent.

Patients who were diagnosed with NSCLC and treated in Sun Yat-sen University Cancer Center between May 2012 and Feb 2019 were retrospectively reviewed. Cases was examined to meet the following criteria: 1. Pathologically confirmed pulmonary LELC; 2. Stage I–III disease according to American Joint Committee on Cancer/Union for International Cancer Control 8th staging criteria (10), based on results of chest and upper abdominal computed tomography (CT), brain magnetic resonance imaging, and bone scan. Positron emission tomography (PET)/CT was allowed as a substitute for the extracranial radiologic work-ups; 3. Either initially diagnosed or locally recurrent disease was included; 4. Patients were treated by local therapy (surgery or radiation) with curative intent, with or without systematic approach; 5. Plasma EBV-DNA copy number was tested around the treatment (pre- or post-treatment EBV-DNA was tested within 1-month before or 2 months after the treatment, respectively) and/or during follow-up (2 months after the completion of treatment). Patients with incomplete clinic-pathologic data or clinical follow-up <6 months were excluded.

### Treatments

Local therapy, either surgery or radiotherapy, was delivered to the primary or locally recurrent disease, with curative intent. The selection of treatment depended on the stage, size, and location of disease, previous treatments, patient status, and comorbidities.

For those receiving radical thoracic surgery, lobectomy was most commonly performed. In some cases, pneumonectomy was performed after fully assessment by the multidisciplinary team to ensure the balance of survival benefit and the preservation of lung functions. Ipsilateral hilar and mediastinal lymph node dissection was a routine.

Patients receiving radical, adjuvant, or salvage chest radiotherapy were immobilized in a vacuum pad supine, and scanned with a CT slice thickness of 5 mm. The respiration motion was obtained by performing four-dimensional CT scanning. The maximum intensity projection images were reconstructed by combing the images collected in 10 phases of respiratory cycle. The gross tumor volume (GTV) was delineated restricted to the traceable primary tumor and metastatic lymph nodes on each phase of the four-dimensional CT. The clinical target volume (CTV) was contoured to cover 6 mm surrounding the GTV and positive node regions, as well as elective regions in some cases, decided by individual radiation oncologist. PTV-GTV and PTV-CTV was created by expanding GTV and CTV with a 0.6 cm margin in all directions, respectively. For radical/salvage irradiation, a total of 60–70 Gy was prescribed to PTV-GTV, and 40–50 Gy to PTV-CTV. For post-operative radiation therapy, 50 Gy was prescribed to PTV-CTV. Either three-dimensional conformal radiation therapy or intensity modulated radiation therapy technique was used for treatment planning and delivery. Patients received radiotherapy once daily.

For patients with locally advanced or recurrent disease, chemotherapy was usually delivered for at least four cycles. The regimens were combinations of taxols/pemetrexed/gemcitabine and platinum. In patients with poor performance status, oral fluorouracil was preferred. Concurrent chemotherapy (taxols/pemetrexed and platinum) was administrated with radiotherapy if there was any sign of radiologic visible tumor.

### Plasma EBV-DNA Testing

Peripheral blood (7.5 mL) was obtained from each patient, and collected in an EDTA tube (Becton Dickinson), centrifuged at 3,000 × g for 10 min. The plasma EBV-DNA was extracted by a QIAamp DNA Mini Kit (Qiagen). The extract amount was record to calculate the targeted DNA concentration. The EBV-DNA extracted from the column was eluted in 50 μL distilled water.

The real-time quantitative polymerase chain reaction (PCR) system was used for plasma EBV-DNA testing toward the BamHI-W region. The primer sequence was forward, 5′-AGTCTCTGCCTCAGGGCA-3′; and reverse, 5′-ACAGAGGGCCTGTCCACCG-3′. The sequence of the probe, a dual fluorescence-labeled oligomer, was 5′-[FAM] CACTGTCTGTAAAGTCCAGCCTCC [TAMRA]-3′. The sequence data for the EBV genome was obtained from the GeneBank sequence data base with the accession number V01555 (11). Quantitative PCR for the β-actin gene served as a control for the amplifiability of circulating DNA. The primer sequence was as follows: forward, 5′-ACAGGCACCAGGGCGTGATGG-3′; and reverse, 5′-CTCCATGTCG TCCCAGTTGGT-3′. The dual-labeled fluorescent probe sequence was 5′-[FAM] CATCCTCACCCTGAAGTACCCCA TC [TAMRA]-3′.

Multiple water blanks were used in the analysis as negative control. The copy number of the plasma EBV genome per mL was record. To ensure the reliability, each sample was tested for twice, and the mean quantity was used for the calculation of EBV-DNA copy number. The real-time quantitative PCR and reaction set-up procedures were described previously by Shao et al. (11).

### Follow-Up

Regular follow-up started 1–2 months after the end of local treatment. The clinical work-ups included chest and upper abdominal CT, and brain magnetic resonance imaging, every 3–6 months. PET/CT and biopsy were performed if necessary. Tumor response was evaluated according to Response Evaluation Criteria in Solid Tumor 1.1.

### Statistical Methods

Progression-free survival (PFS) and overall survival (OS) was defined as the time from treatment start to first progressive disease or death, or to death from any cause, respectively. Any failure appeared in the ipsilateral hemithorax (except for newly found multiple lesions in the ipsilateral lung), or the regional lymph nodes was regarded as a locoregional progression; otherwise it was considered as a distant metastasis. The time from treatment start to first locoregional progression or distant metastasis was recorded to calculate locoregional progression-free survival and distant metastasis-free survival, respectively.

The distribution of categorical variables was assessed by Fisher's exact test. The Kaplan-Meier method and log-rank test were used to describe and compare the survival, respectively. Univariable Cox proportional hazard model was applied to calculate the hazard ratios. Any factor with *p* < 0.10 in the univariable analysis was incorporated in the multivariable Cox proportional hazards model. Logistic regression model was used for multivariable analysis of categorical variables. *P* < 0.05 (two-sided) were considered of statistical significance. Statistics was conducted using SPSS 22.0 (IBM Corp.).

## Results

A total of 102 patients met the study criteria and were included in analysis. Four datasets were analyzed, including patients with pre-treatment (*N* = 66), post-treatment (*N* = 93) and follow-up (*N* = 58) and complete (*N* = 41, pre-treatment, post-treatment, and at least one follow-up test) plasma EBV-DNA results, respectively (Supplementary Figure 1). Patients in the follow-up set had post-treatment EBV-DNA results and at least one extra EBV-DNA test before the confirmation of disease progression. EBV-DNA was tested at a median time of 8 (range, 1–20) and 48 (range: 1–60) days before or after treatment, respectively. Table 1 presented the clinic-pathologic and treatment-related characteristics of the whole cohort and patients in each dataset. Overall, the median follow-up time was 29 (range, 6–80) months.

**Table 1:** Patient characteristics.

**Variable**	**All patients**	**Pre-treatment set**	**Post-treatment set**	**Follow-up set**	**Full data set**
	***N =* 102 (%)**	***N =* 66 (%)**	***N =* 93 (%)**	***N =* 58 (%)**	***N =* 41 (%)**
Sex					
Male	44 (43.1)	30 (45.5)	41 (44.1)	25 (43.1)	20 (48.8)
Female	58 (56.9)	36 (54.5)	52 (55.9)	33 (56.9)	21 (51.2)
Age					
<50	44 (43.1)	26 (39.4)	39 (41.9)	25 (43.1)	15 (36.6)
≥ 50	58 (56.9)	40 (60.6)	54 (58.1)	33 (56.9)	26 (63.4)
Smoking					
Yes	26 (25.5)	63 (95.5)	26 (28.0)	13 (22.4)	10 (24.4)
No	76 (74.5)	3 (4.5)	67 (72.0)	45 (77.6)	31 (75.6)
Chemotherapy					
Yes	74 (72.5)	52 (78.8)	79 (84.9)	51 (87.9)	39 (95.1)
No	28 (27.5)	14 (21.2)	14 (15.1)	7 (12.1)	2 (4.9)
Local therapy					
Surgery + RT	14 (13.7)	5 (7.6)	14 (15.1)	10 (17.2)	4 (9.8)
Surgery	33 (32.4)	12 (18.2)	32 (34.4)	13 (22.4)	6 (14.6)
RT	55 (53.9)	49 (74.2)	47 (50.5)	35 (60.3)	31 (75.6)
Status					
Initial diagnosis	88 (86.3)	53 (80.3)	82 (88.2)	49 (84.5)	33 (80.5)
Recurrent disease	14 (13.7)	13 (19.7)	11 (11.8)	9 (15.5)	8 (19.5)
Stage					
I	18 (17.6)	9 (13.6)	17 (18.3)	11 (19.0)	7 (17.1)
II	13 (12.7)	6 (9.1)	12 (12.9)	5 (8.6)	3 (7.3)
III	71 (69.6)	51 (77.3)	64 (68.8)	42 (72.4)	31 (75.6)
Pre-treatment EBV-DNA (copies/mL)					
≤ 10,000	–	35 (53.0)	–	–	24 (58.5)
>10,000	–	31 (47.0)	–	–	17 (41.5)
Post-treatment EBV-DNA (copy/mL)					
0	–	–	74 (79.6)	–	34 (82.9)
>0	–	–	19 (20.4)	–	7 (17.1)
EBV-DNA during follow-up					
Increasing	–	–	–	19 (32.8)	15 (36.6)
Not increasing	–	–	–	39 (67.2)	26 (63.4)
EBV-DNA during follow-up					
Increasing and > 1,000 copies/mL	–	–	–	10 (17.2)	7 (17.1)
Not increasing or ≤ 1,000 copies/mL	–	–	–	48 (82.8)	34 (82.9)

*RT, radiotherapy; EBV, Epstein-Barr virus; DNA, deoxyribonucleic acid*.

### PFS and OS

At the last follow-up, 33 patients had disease progression. A total of 6 deaths were documented. The 2-year PFS and OS were 66.3% (95% CI, 56.5–76.1%) and 96.0% (95% CI, 91.5–100.5%), respectively.

### Patterns of Recurrence and Salvage Treatments

Among the patients with disease progression, there were 15 (14.7%) with local recurrence only, 15 (14.7%) with distant metastases only, and 2 (2.0%) with both. The 2-year locoregional progression-free survival and distant metastasis-free survival were 83.7% (95% CI, 76.1–91.3%) and 81.3% (95% CI, 73.3–89.3%), respectively.

After the first failure, 26 patients (81.2%) received salvage treatments including: chemotherapy alone (*n* = 11), chemotherapy + radiotherapy (*n* = 8), TKI alone (*n* = 2), microwave ablation or cryoablation (*n* = 4) and surgery + chemotherapy (*n* = 1). The other six patients had traditional Chinese medicine or palliative therapy.

### Pre-treatment Set

**Figure 2:**
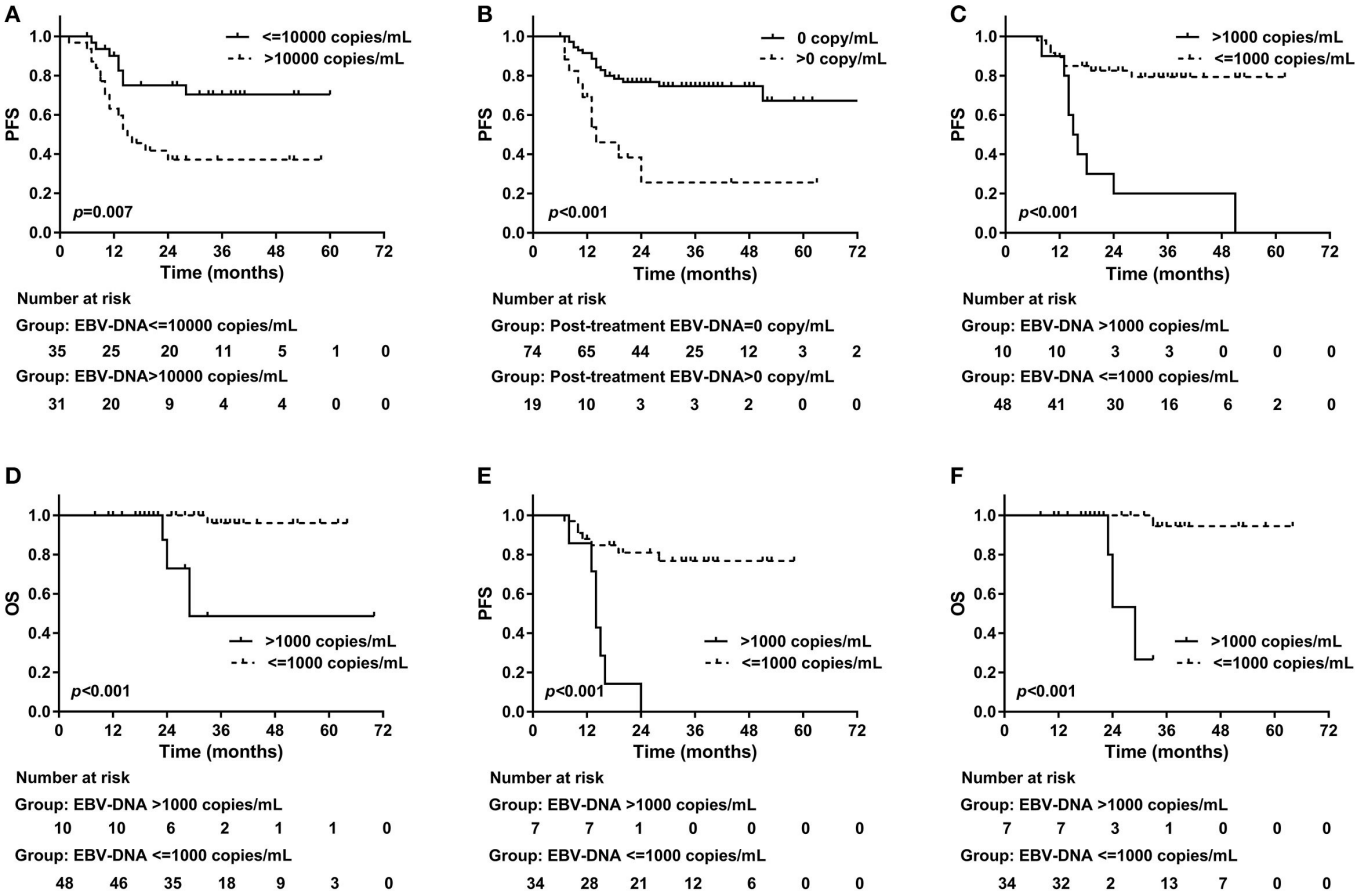
Progression-free survival and plasma EBV-DNA results. **(A)** Pre-treatment plasma EBV-DNA copy number > 10,000 indicated an impaired 2-year PFS (> 10,000 vs. ≤ 10,000 copies/mL, 37.2 vs. 75.1%, *p* = 0.007, log-rank); **(B)** Positive post-treatment plasma EBV-DNA was associated with worse 2-year PFS (> 0 vs. 0 copy/mL, 25.6 vs. 76.8%, *p* < 0.001, log-rank). During follow-up, an increasing EBV-DNA with the copy number exceeding 1,000 copies/mL predicted worse **(C)** 2-year PFS (> 1,000 copies/mL vs. ≤ 1,000 copies/mL, 20.0 vs. 82.5%, *p* < 0.001, log-rank) and **(D)** 2-year OS (> 1,000 copies/mL vs. ≤ 1,000 copies/mL, 72.9 vs. 100%, *p* < 0.001, log-rank). In patients with complete EBV-DNA results, an increasing EBV-DNA with the copy number exceeding 1,000 copies/mL predicted worse **(E)** 2-year PFS (> 1,000 copies/mL vs. ≤ 1,000 copies/mL, 0 vs. 81.0%, *p* < 0.001, log-rank) and **(F)** 2-year OS (> 1,000 copies/mL vs. ≤ 1,000 copies/mL, 53.3 vs. 100%, *p* < 0.001, log-rank). EBV, Epstein-Barr virus; DNA, deoxyribonucleic acid; PFS, Progression-free survival; OS, overall survival.

Among all of the 66 patients presenting pre-treatment EBV-DNA results, 59 (89.4%) showed detectable plasma EBV-DNA. The median copy number was 8,685 copies/mL, with a range from 0 to 2,010,000 copies/mL. Twenty-five patients were found of disease progression and six died. The median follow-up time of this set was 25 (range, 6–72) months. The 2-year PFS and OS were 56.2% (95% CI, 43.1–69.3%) and 93.0% (95% CI, 85.4–100.6%), respectively. Table 2 showed the results of univariable and multivariable analyses of clinical outcomes. Pre-treatment EBV-DNA copy number exceeding 10,000 predicted a poor 2-year PFS (>10,000 vs. ≤ 10,000 copies/mL, 37.2 vs. 75.1%, *p* = 0.007, log-rank, Figure 2A). None of the clinic-pathologic features showed significant statistical relation with OS.

**Table 2:** Univariable Cox regression analysis in the pre-treatment set.

**Variable**	**Univariable analysis**			
	**PFS**			**OS**
	**HR**	**95% CI**	***p***	***p***
Sex (male vs. female)	1.03	0.49–2.17	*0.938*	*0.207*
Age (<50 vs. ≥ 50)	1.02	0.42–2.47	*0.965*	*0.325*
Smoking (Yes vs. No)	0.45	0.15–1.32	*0.147*	*0.331*
Chemotherapy (Yes vs. No)	22.69	0.028–1.79E04	*0.359*	*0.618*
Local therapy (Surgery + RT vs. Surgery vs. RT)	–	–	*0.944*	*0.403*
Surgery + RT vs. RT	0.78	0.19–3.29	*0.735*	*0.988*
Surgery vs. RT	0.99	0.47–2.09	*0.979*	*0.514*
Status (Initial diagnosis vs. Recurrent disease)	1.99	0.60–6.62	*0.262*	*0.429*
Stage (I–II vs. III)	0.41	0.12–1.37	*0.147*	*0.402*
Pre-treatment EBV-DNA (≤ 10,000 vs. > 10,000 copies/mL)	0.34	0.15–0.78	***0.011***	*0.293*

PFS, Progression-free survival; OS, overall survival; HR, hazard ratio; CI, confidence interval; RT, radiotherapy; EBV, Epstein-Barr virus; DNA, deoxyribonucleic acid.

*HR and CI were not calculated in the univariable analysis for OS because the number of death was limited. Bold values indicates p values < 0.05. All p-values are shown italic*.

### Post-treatment Set

EBV-DNA was still detectable after treatment in 19 of 93 (20.4%) patients. The median post-treatment EBV-DNA copy number was 0 (range, 0–1010000) copy/mL. EBV-DNA copy number decreased in all patients after treatment, where pre-treatment EBV-DNA results were available (*N* = 57). The median follow-up time was 29 (range, 6–80) months. At last follow-up, there were 27 patients diagnosed with progressive disease, and 5 deaths were reported. The 2-year PFS and OS were 68.7% (95% CI, 58.7–78.7%) and 95.7% (95% CI, 90.8–100.6%), respectively. Patients with stage III disease were associated with higher risk of disease progression compared with those with stage I–II (2-year PFS, stage I–II vs. III, 88.6 vs. 59.5%, *p* = 0.021, log-rank) or with chemotherapy (2-year PFS, chemotherapy vs. without chemotherapy, 62.2 vs. 100%, *p* = 0.006, log-rank). Patients treated with chemotherapy (60/79, 75.9%) was significantly more likely to have stage III disease compared with those without chemotherapy (4/14, 28.6%, *p* = 0.001). If post-treatment EBV-DNA was still detectable, the disease was more likely to recur within 2 years as well (2-year PFS, >0 vs. 0 copy/mL, 25.6 vs. 76.8%, *p* < 0.001, log-rank, Figure 2B). Positive post-treatment EBV-DNA remained as the only independent risk factor in the multivariable analysis on PFS (HR = 3.44, 95% CI, 1.52–7.78; *p* = 0.003). Stage III disease showed marginal significance in the prediction of OS (2-year OS, stage I–II vs. III, 100 vs. 93.5%, *p* = 0.081, log-rank) (Table 3).

**Table 3:** Univariable and multivariable Cox regression analyses in the post-treatment set.

**Variable**	**PFS**						**OS**
	**Univariable analysis**			**Multivariable analysis**			**Univariable analysis**
	**HR**	**95% CI**	***p***	**HR**	**95% CI**	***p***	***p***
Sex (male vs. female)	0.90	0.19–4.37	*0.896*				*0.278*
Age (<50 vs. ≥50)	0.51	0.23–1.15	*0.105*				*0.489*
Smoking (Yes vs. No)	0.56	0.23–1.37	*0.203*				*0.355*
Chemotherapy (Yes vs. No)	29.41	1.29–670.06	***0.034***				*0.468*
Local therapy (Surgery + RT vs. Surgery vs. RT)	–	–	*0.191*				*0.425*
Surgery + RT vs. RT	0.62	0.21–1.83	*0.387*				*0.981*
Surgery vs. RT	0.45	0.18–1.10	*0.079*				*0.191*
Status (Initial diagnosis vs. Recurrent disease)	1.56	0.37–6.63	*0.547*				*0.592*
Stage (I–II vs. III)	0.31	0.11–0.89	***0.030***				***0.023***
Post-treatment EBV-DNA (>0 copy/mL vs. 0)	3.97	1.75–9.03	***0.001***	3.44	1.52–7.78	***0.003***	*0.600*

PFS, Progression-free survival; OS, overall survival; HR, hazard ratio; CI, confidence interval; RT, radiotherapy; EBV, Epstein-Barr virus; DNA, deoxyribonucleic acid.

*HR and CI were not calculated in the univariable analysis for OS because the number of death was limited. Bold values indicates p values < 0.05. All p-values are shown italic*.

### Follow-Up Set

In this dataset, all patients had post-treatment EBV-DNA result and at least one extra EBV-DNA test before the confirmation of first disease progression. Of the 58 patients included in this set, the median follow-up time was 31 (range, 8–70) months. The patients had EBV-DNA tests with a median of 4 (range, 1–10) times since 2 months after the treatment, and before disease progression or death. In 3 patients, EBV-DNA was only tested immediately before the detection of relapse. Twenty-two patients were found of progressive disease (PD) and four deaths were recorded. The overall 2-year PFS and OS were 70.6% (95% CI, 58.4–82.8%) and 95.2% (95% CI, 88.7–101.7%), respectively. Based on the post-treatment EBV-DNA result, 19 (32.8%) patients presented increasing EBV-DNA copy numbers during follow-up. Ten (17.2%) showed increasing EBV-DNA level with the number exceeding 1,000 copies/mL (excluding patients with persistent EBV-DNA level > 1,000 copies/mL after treatment but with a declined tendency during follow-up). Twelve PD patients showing increasing EBV-DNA copy number, 8 had disease progression within 3 months after the detection of plasma EBV-DNA. The other four were found of PD at 5, 7, 10, and 17 months later, respectively. Positive post-treatment EBV-DNA was related with poorer 2-year PFS (> 0 vs. 0 copy/mL, 18.2 vs. 77.6%, *p* = 0.002, log-rank). The increase of EBV-DNA copy number suggested an impaired 2-year PFS (increasing vs. not increasing EBV-DNA, 37.7 vs. 86.8%, *p* < 0.001, log-rank). Using the increase of EBV-DNA copy number as an indicator of immediate disease progression within 3 months, the specificity was 75.0% (95% CI, 59.7–86.8%), and the sensitivity was 57.2% (95% CI, 28.9–82.2%). Among the ten patients with increasing EBV-DNA copy number and had a result > 1,000 copies/mL, 9 of them had disease progression, 8 within 3 months, and 1 at 17 months later. PD was also more frequently found in those with a climbing EBV-DNA level exceeding 1,000 copies/mL (2-year PFS, > 1,000 vs. ≤ 1,000 copies/mL, 20.0 vs. 82.5%, *p* < 0.001, log-rank, Figure 2C), as well as death (2-year OS, > 1,000 vs. ≤ 1,000 copies/mL, 72.9 vs. 100%, *p* < 0.001, log-rank, Figure 2D). The association between PD and increasing EBV-DNA over 1,000 copies/mL remained statistically significant in multivariable analysis (HR = 5.08; 95% CI, 1.93–13.38; *p* = 0.001, Table 4). Using 1,000 copies/mL as the cut-off value, the specificity and sensitivity for disease relapse within 3 months were 97.5% (95% CI, 86.8–99.6%) and 50.0% (95% CI, 26.1–73.9%) (Figure 1C).

**Table 4:** Univariable and multivariable Cox regression analyses in the follow-up set.

**Variable**	**PFS**						**OS**
	**Univariable analysis**			**Multivariable analysis**			**Univariable analysis**
	**HR**	**95% CI**	***p***	**HR**	**95% CI**	***p***	***p***
Sex (male vs. female)	0.87	0.35–2.18	*0.766*				*0.366*
Age (<50 vs. ≥50)	0.44	0.16–1.24	*0.119*				*0.295*
Smoking (Yes vs. No)	0.36	0.08–1.57	*0.174*				*0.500*
Chemotherapy (Yes vs. No)	26.32	9.55E−2-7.26E3	*0.254*				*0.538*
Local therapy (Surgery + RT vs. Surgery vs. RT)	–	–	*0.441*				*0.628*
Surgery + RT vs. RT	0.44	0.10–1.95	*0.279*				*0.527*
Surgery vs. RT	0.58	0.16–2.05	*0.398*				*0.464*
Status (Initial diagnosis vs. Recurrent disease)	1.49	0.34–6.46	*0.594*				*0.533*
Stage (I–II vs. III)	0.49	0.14–1.70	*0.261*				*0.409*
Post-treatment EBV-DNA (> 0 copy/mL vs. 0)	4.55	1.58–13.11	***0.005***				*0.743*
EBV-DNA during follow-up (Increasing vs. Not increasing)^*^	5.26	1.96–14.14	0.001^**^				*0.060*
EBV-DNA during follow-up (Increasing and > 1,000 copies/mL vs. Not increasing or ≤ 1,000 copies/mL)	6.17	2.40–15.85	***<0.001***	5.08	1.93–13.38	***0.001***	***0.008***

PFS, Progression-free survival; OS, overall survival; HR, hazard ratio; CI, confidence interval; RT, radiotherapy; EBV, Epstein-Barr virus; DNA, deoxyribonucleic acid.

* Patients with persistent increased EBV-DNA level > 1,000 copies/mL after treatment but with a declined tendency were not included.

** Although showing predictive value in univariable analysis, the parameter was not included in multivariable analysis because of its multicollinearity with EBV-DNA during follow-up (Increasing and > 1,000 copies/mL vs. Not increasing or ≤ 1,000 copies/mL).

*HR and CI were not calculated in the univariable analysis for OS because the number of death was limited. Bold values indicates p values < 0.05. All p-values are shown italic*.

### Full Data Set

There were 41 patients with results of all pre-treatment, post-treatment and at least one follow-up EBV-DNA. The characteristics of these patients are presented in Table 1. The patients were tested for EBV-DNA with a median of 4 (range, 1–10) times during follow-up. One patient had EBV-DNA test only immediately before the detection of relapse. Until last follow-up, 14 patients were found of PD and four deaths were recorded. The 2-year PFS and OS were 65.3% (95% CI, 49.8–80.8%) and 92.4% (95% CI, 82.4–102.4%), respectively. Fifteen (36.6%) patients showed increasing EBV-DNA level during follow-up and 7 (17.1%) had increasing EBV-DNA copy number exceeding 1,000 copies/mL. Among the 10 PD patients with increasing EBV-DNA copy number, 8 had disease progression within 3 months, and the other 2 were confirmed of disease progression at 5 and 7 months later, respectively. All of the 7 patients with increasing EBV-DNA level > 1,000 copies/mL had disease progression within 3 months.

In univariable analysis, there was a trend toward a worse 2-year PFS in patients with pre-treatment EBV-DNA > 10,000 copies/mL, but without statistical significance (> 10,000 vs. ≤ 10,000 copies/mL, 49.9 vs. 77.0%, *p* = 0.190, log-rank). Post-treatment EBV-DNA>0 was a risk factor of PFS (> 0 vs. 0 copy/mL, 0 vs. 76.0%, *p* < 0.001, log-rank), as well as the increase of EBV-DNA copy number (increasing vs. not increasing EBV-DNA, 26.0 vs. 88.1%, *p* < 0.001, log-rank) and increasing EBV-DNA copy number over 1,000 copies/mL (> 1,000 vs. ≤ 1,000 copies/mL, 0 vs. 81.0%, *p* < 0.001, log-rank, Figure 2E). EBV-DNA copy number > 1,000 copies/mL was the only independent risk factor of PFS in multivariate analysis (HR = 5.46, 95% CI, 1.53–19.51; *p* = 0.008). Identified in univariate analysis, the factors predicting a worse 2-year OS were the increase of EBV-DNA copy number (increasing vs. not increasing EBV-DNA, 76.2 vs. 93.3%, *p* = 0.027, log-rank) and increasing EBV-DNA copy number over 1,000 copies/mL (> 1,000 vs. ≤ 1,000 copies/mL, 53.3 vs. 100%, *p* < 0.001, log-rank, Figure 2F). None of the factors independently predicted OS in the multivariable analysis (Table 5).

**Table 5:** Univariable and multivariable Cox regression analyses in the full data set.

	**PFS**						**OS**
	**Univariable analysis**			**Multivariable analysis**			**Univariable analysis**
	**HR**	**95% CI**	***p***	**HR**	**95% CI**	***p***	***p***
Sex (male vs. female)	0.82	0.28–2.43	*0.720*				*0.336*
Age (<50 vs. ≥ 50)	0.64	0.20–2.03	*0.448*				*0.268*
Smoking (Yes vs. No)	0.19	0.03–2-1.45	*0.109*				*0.473*
Chemotherapy (Yes vs. No)	22.73	2.05E−3-2.52E5	*0.511*				*0.685*
Local therapy (Surgery + RT vs. Surgery vs. RT)	–		*0.551*				*0.773*
Surgery + RT vs. RT	0.52	0.07–3.91	*0.525*				*0.643*
Surgery vs. RT	0.38	0.05–2.88	*0.349*				*0.582*
Status (Initial diagnosis vs. Recurrent disease)	1.68	0.38–7.51	*0.497*				*0.450*
Stage (I–II vs. III)	0.46	0.10–2.09	*0.315*				*0.450*
Pre-treatment EBV-DNA (≤ 10,000 vs. > 10,000 copies/mL)	0.49	0.17–1.41	*0.187*				*0.458*
Post-treatment EBV-DNA (> 0 copy/mL vs. 0)	5.99	1.92–18.64	*0.002*				*0.776*
EBV-DNA during follow-up (Increasing vs. Not increasing)^*^	6.02	1.84–19.70	0.003^**^				***0.065***
EBV-DNA during follow-up (Increasing and > 1,000 copies/mL vs. Not increasing or ≤ 1,000 copies/mL)	8.20	2.65–25.36	***<0.001***	5.46	1.53–19.51	***0.008***	***0.005***

PFS, Progression-free survival; OS, overall survival; HR, hazard ratio; CI, confidence interval; RT, radiotherapy; EBV, Epstein-Barr virus; DNA, deoxyribonucleic acid.

* Patients with persistent increased EBV-DNA level > 1,000 copies/mL after treatment but with a declined tendency were not included.

** Although showing predictive value in univariable analysis, the parameter was not included in multivariable analysis because of its multicollinearity with EBV-DNA during follow-up (Increasing and > 1,000 copies/mL vs. Not increasing or ≤ 1,000 copies/mL).

*HR and CI were not calculated in the univariable analysis for OS because the number of death was limited. Bold values indicates p values < 0.05. All p-values are shown italic*.

If the increase of EBV-DNA copy number was used to predict immediate disease progression, the specificity was 75.9% (95% CI, 56.5–89.7%), and the sensitivity was 66.7% (95% CI, 34.9–89.9%). When 1,000 copies/mL was used as the cut-off value, the specificity and sensitivity for disease progression within 3 months were 100% (95% CI, 76.7–100%) and 50.0% (95% CI, 23.1–76.9%).

**Figure 3:**
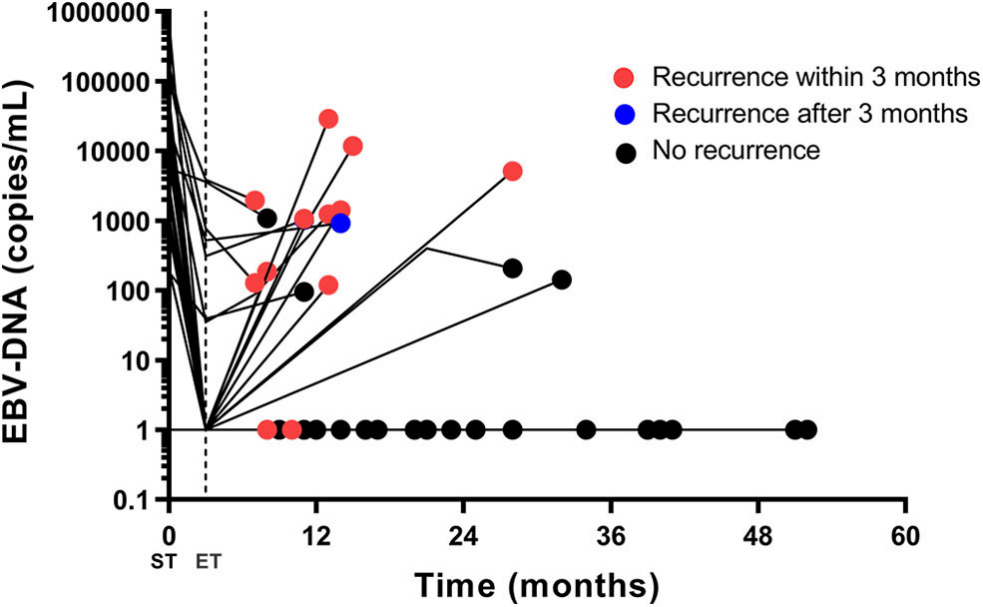
The EBV-DNA trajectory for the 41 patients having pre-treatment, post-treatment, and follow-up results. Pre-treatment, post-treatment and at least one follow-up EBV-DNA (first increase and first increasing EBV-DNA level exceeding 1,000 copies/mL) results are plotted. If an EBV-DNA copy number declines or remains unchanged during follow-up, only the last EBV-DNA copy number is plotted. The red dot stands for radiologic recurrence within 3 months, and the blue dot stands for radiologic recurrence after 3 months. The black dot stands for no radiologic recurrence. Most of the patients with persistent EBV-DNA copy number = 0 are free of recurrence. All patients with an increasing EBV-DNA > 1,000 copies/mL had immediate disease recurrence. EBV, Epstein-Barr virus; DNA, deoxyribonucleic acid; ST, start of treatment; ET, end of treatment.

The EBV-DNA trajectory for the 41 patients including representative EBV-DNA point data was detailed in Figure 3. Most of the patients with persistent EBV-DNA copy number = 0 are free of recurrence. All patients with an increasing EBV-DNA > 1,000 copies/mL had immediate disease recurrence.

## Discussion

We retrospectively investigated the prognostic effect of EBV-DNA value on treatment response and disease development of pulmonary LELC. In our cohort, the overall 2-year PFS and OS was 66.3 and 96.0%, respectively, suggesting a much favorable prognosis of pulmonary LELC compared with other common types of lung cancers, in consistent with previous reports (1–2, 5–6). Approximately 90% of patients were detected with pre-treatment positive EBV-DNA, reflecting an intimate relationship between EBV and pulmonary LELC. Similarly, Han et al. (8) found that 94% of all pulmonary LELC cases showed positive EBER in tumor cells, with 53.5 and 23.3% expressed latent membrane protein 1 and viral capsid antigen, respectively, while none of non-LELC lung carcinoma expressed any of the viral markers.

Circulating EBV-DNA in plasma is derived from apoptotic or necrotic tumor cells. The dynamic change of plasma EBV-DNA is a reflection of the tumor burden. Our results supported that plasma EBV-DNA should be examined routinely and regularly in pulmonary LELC. Either pre-treatment EBV-DNA over 10,000 copies/mL, or positive post-treatment EBV-DNA predicted a worse PFS but not OS. A similar study performed by Xie et al. (4) explored the clinical significance of plasma EBV-DNA copy number in 429 patients with stage I-IV pulmonary LELC. Baseline EBV-DNA copy value over 4,000 copies/mL represented a significant increased risk of death. In 309 patients with stage I-III pulmonary LELC treated with radical resection, those with persistently detectable plasma EBV-DNA showed significantly worse OS and DFS. The possible explanation for the inconsistent results was that our study only included patients diagnosed with local disease and radical treatments were performed for all traceable lesions. The poor prognosis might be reversed by the intensive therapies. The hypothesis was furtherly supported by our data that both PFS and OS could be predicted by significant increase of follow-up EBV-DNA level (> 1,000 copies/mL). In sum, for patients with local disease and curative treatment, a regular test of EBV-DNA is essential for the prognosis and monitoring of pulmonary LELC.

Xie et al. (4) suggested a significant relation between EBV-DNA change and disease burden in advanced disease (4), however, the exact cut-off value of EBV-DNA load predicting tumor progression in the near future was not specified, thus it was still difficult to explain the EBV-DNA results obtained in the follow-up period. We analyzed patients with post-treatment EBV-DNA result and at least one extra test during follow-up, before the occurrence of disease progression. The median time for extra EBV-DNA testing in these patients was four. Although a simple increase of EBV-DNA copy number could hardly give an accurate prediction of disease progression, an increasing plasma EBV-DNA copy number exceeding 1,000 copies/mL during follow-up strongly implied a chance of immediate radiologic recurrence, with a specificity of 97.5–100%, or a false positive rate of 0–2.5%. Intensive radiologic work-ups will be important in these patients. Importantly, false negatives were often noticed, suggesting that a low EBV-DNA did not necessarily ensure disease control.

In the post-treatment set, chemotherapy seemed to be a risk factor of PFS. The reason was that patients with stage I disease was less likely to receive chemotherapy, and those with stage I-II disease had significantly favored PFS. Chemotherapy did not remain an independent risk factor in multivariable analysis.

In general, potential biomarkers in LELC could be derived from EBV, malignant cells or specific body response to either virus or the tumor. Plasma EBV-DNA, as a virus-related indicator, has been highly emphasized due the simple acquisition and low expense. Other options, such as viral messenger RNAs and C-promoter region methylation status of EBV in cytology specimens, were also proven effective for minimally invasive NPC diagnosis (12) but have not put into routine use. Circulating tumor cells have been studied as a tumor-dependent biomarker. Despite of an acceptable sensitivity (75%) and specificity (92%) for lung cancer diagnosis (13), and an established role in disease prognosis (14–17), methods for the detection of circulating tumor cells were still inconsistent and the result interpretation was not standardized (18). Other researchers focused on the host-relative biomarkers. Wang et al. (19) demonstrated that pre-treatment monocyte-to-lymphocyte ratio over 0.262 as an independent risk factor of PFS and OS in pulmonary LELC. Other informative assessments, e.g., radiologic imaging, were vulnerable to the cost, radiation exposure, and difficulty in differentiating recurrence and post-treatment change. A recent study revealed that pretreatment [11F]-2-fluoro-2-deoxy-D-glucose PET assisted in the staging of pulmonary LELC, and was an independent prognostic factor for OS (20).

Our knowledge of pulmonary LELC used to be limited. One comparative study brought inspiring insights. Hong et al. investigated the mutational landscape of pulmonary LELC via whole-exome sequencing, targeted deep sequencing, as well as single-nucleotide polymorphism arrays, and compared it to those of NPC and other lung cancers. It turned out that pulmonary LELC resembled NPC, rather than other lung cancers in multiple genetic features (21). These findings supported that plenty of experience could be learned from NPC, where the role of EBV-DNA has been well-established for prognosis, surveillance, and even disease screening (22). For instance, sub-classification of disease by the size profile differences of EBV-DNA was possible (23). Several EBV-encoded latent proteins were proved to mediate cellular transformation and contributed in carcinogenesis (24). Ephrin receptor A2 was found to be an epithelial cell receptor for EBV entry (25). Such valuable data might be extracted for the future exploration of pulmonary LELC.

The current study has several limits. First, the rarity of pulmonary LELC is an obstacle of collecting large scale of homogenous data. It is inevitable to analyze patients with relatively different stage and inconsistent treatments. Patients with either initial diagnosed disease or local recurrence was analyzed together, however, radical treatment was a mandatory inclusion criterion. Second, the detection EBV-DNA copy number was not a clinical routine, while missing data might cause bias. Patients received extra EBV-DNA testing at a median of four times during follow-up. Three patients were only sent for EBV-DNA test because there was clinical hint of disease progression. A further analysis focusing on the short-term, dynamic change EBV-DNA in such cases could be helpful to determine the best cut-off value of EBV-DNA for disease monitoring.

## Conclusions

In patients receiving radial local treatments for stage I–III, primary or locally recurrent pulmonary lymphoepithelioma-like carcinoma, pre- and post- treatment and follow-up plasma EBV-DNA levels predict the progression-free survival. An increasing EBV-DNA copy number exceeding 1,000 copies/mL during follow-up predicts OS, and is strongly related to disease progression within 3 months. Regular testing of EBV-DNA is suggested for pulmonary LELC. If an EBV-DNA copy number increased and was found beyond 1,000 copies/mL during follow-up, intensive radiologic evaluations are recommended.

## Data Availability Statement

The raw data supporting the conclusions of this article will be made available by the authors, without undue reservation.

## Ethics Statement

The studies involving human participants were reviewed and approved by Ethics committee of Sun Yat-sen University Cancer Center. Written informed consent for participation was not required for this study in accordance with the national legislation and the institutional requirements.

## Author Contributions

HL and M-SZ contributed to conception and design of the study. Q-WL, W-MH, S-PG, Y-JW, Y-JZ, NH, X-LA, N-BC, J-YG, Y-HH, and M-ZL organized the database. Q-WL performed the statistical analysis. Q-WL and BQ wrote the first draft of the manuscript. All authors contributed to manuscript revision, read, and approved the submitted version.

## Conflict of Interest

The authors declare that the research was conducted in the absence of any commercial or financial relationships that could be construed as a potential conflict of interest.
