# Supplementary figures and images for: Plasma Epstein-Barr Virus-Deoxyribonucleic Acid Copy Number Predicts Disease Progression in Stage I–III Pulmonary Lymphoepithelioma-Like Carcinoma
Source: Front Oncol. 2020 Aug 21;10:1487. doi: 10.3389/fonc.2020.01487 (PMC7473393; doi:10.3389/fonc.2020.01487)

Supplementary Fig.1 Number of patients in each dataset

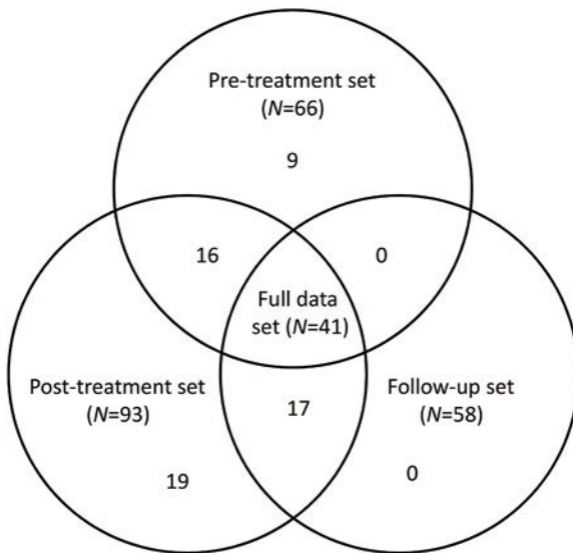

Supplement: Supplementary file 1 [file Image_1.pdf]
